# Prediction of Drought-Resistant Genes in *Arabidopsis thaliana* Using SVM-RFE

**DOI:** 10.1371/journal.pone.0021750

**Published:** 2011-07-15

**Authors:** Yanchun Liang, Fan Zhang, Juexin Wang, Trupti Joshi, Yan Wang, Dong Xu

**Affiliations:** 1 Key Laboratory of Symbol Computation and Knowledge Engineering of Ministry of Education, College of Computer Science and Technology, Jilin University, Changchun, China; 2 Digital Biology Laboratory, Computer Science Department and Christopher S. Bond Life Sciences Center, University of Missouri, Columbia, Missouri, United States of America; University of New Orleans, United States of America

## Abstract

**Background:**

Identifying genes with essential roles in resisting environmental stress rates high in agronomic importance. Although massive DNA microarray gene expression data have been generated for plants, current computational approaches underutilize these data for studying genotype-trait relationships. Some advanced gene identification methods have been explored for human diseases, but typically these methods have not been converted into publicly available software tools and cannot be applied to plants for identifying genes with agronomic traits.

**Methodology:**

In this study, we used 22 sets of *Arabidopsis thaliana* gene expression data from GEO to predict the key genes involved in water tolerance. We applied an SVM-RFE (Support Vector Machine-Recursive Feature Elimination) feature selection method for the prediction. To address small sample sizes, we developed a modified approach for SVM-RFE by using bootstrapping and leave-one-out cross-validation. We also expanded our study to predict genes involved in water susceptibility.

**Conclusions:**

We analyzed the top 10 genes predicted to be involved in water tolerance. Seven of them are connected to known biological processes in drought resistance. We also analyzed the top 100 genes in terms of their biological functions. Our study shows that the SVM-RFE method is a highly promising method in analyzing plant microarray data for studying genotype-phenotype relationships. The software is freely available with source code at http://ccst.jlu.edu.cn/JCSB/RFET/.

## Introduction

Among all kinds of environmental stresses (abiotic and biotic) in worldwide agriculture, drought is a major abiotic stress factor with significant impact on agricultural production. While resistance to biotic stresses is sometimes associated with monogenic traits, abiotic stresses are typically associated with multigenic traits, making it more difficult to study [1]. Hydropenia can trigger a cascade of physiological and metabolic activities in plants so the tolerance and susceptibility to drought are very complex. Among all the plants, *Arabidopsis thaliana* is the most popular model organism used in studying drought tolerance of plants, as it is a typical glycophyte, and many other xerophytes or desiccation-tolerant plants are similar to glycophytes in the drought-resistant mechanism. Hence, we focus on *Arabidopsis* to explore the drought-resistant genes in this study.

Recently, several research groups have investigated drought-mediated changes in gene expression using microarrays [2]–[5]. Microarray datasets typically include several thousands to tens of thousands of genes with relatively a small number of samples, but many genes are irrelevant or redundant for the purpose of this study. Biologically, there are often tens to hundreds of genes significantly associated to a trait like drought resistance. Hence, it is important to develop computational methods to mine these genes based on microarray data.

Prediction of genes associated with a trait can be formulated as a feature selection problem where key features (genes) of microarray data are indicative of a trait. Various feature selection techniques in handling gene expression data have been proposed. In particular, three types of classification-based methods were developed, i.e., filtering methods, wrapper methods, and embedded methods [6]. Feature selection using embedded SVM evaluation criterion to assess feature relevance is a typical and successful method [7]. Several other studies have provided alternative methods. For example, Support Vector Machine-Recursive Feature Elimination (SVM-RFE) was applied to train SVM for obtaining the weight of each feature and removing the one with the smallest weight iteratively [7]–[11]. This algorithm is superior to the “naïve” ranking with only one time RFE [7], [12]. However, this study [13] is the only study to apply the SVM-RFE method for identifying genes of an agronomic trait. Instead, current methods typically use a simple t-test for identifying important genes relevant to a trait [14], [15]. This could be problematic for integrating data from various sources. Furthermore, many researchers currently study genotype-trait relationships in an *ad hoc* fashion, e.g. by manually tuning various parameters that are relatively poorly understood [16]. These issues may result in vast amounts of plant microarray data being underutilized for studying genotype-phenotype relationships. One of the reasons behind these issues is lack of publicly available advanced tools. For example, while the SVM-RFE method is highly promising in analyzing microarray data, there was no software tool available.

In this study, we developed a systematic tool by improving the SVM-RFE method for identifying trait-specific genes using microarray data. The tool characterizes drought-resistant genes in *Arabidopsis thaliana* and is generally applicable to study genotype-phenotype relationships using gene expression data from microarray or RNA-Seq. Furthermore, the tool is freely available with source code at http://ccst.jlu.edu.cn/JCSB/RFET/.

## Materials and Methods

### Data source

GEO (http://www.ncbi.nlm.nih.gov/geo/) currently contains 1664 datasets with 90 GDSs, 299 platforms and 1275 series for *Arabidopsis thaliana*. Among these datasets, we used GSE10670 concerning global expression profiling of wild type and transgenic *Arabidopsis* plants in response to water stress published on September 1, 2008, and last updated on March 15, 2009. From GEO this is the largest gene expression dataset available up to date for studying plant drought resistance. The data was generated from the Affymetrix platform GPL198 *Arabidopsis* ATH1 Genome Array. It consists of 22,810 probes and each probe corresponds to one gene. In all we identified 22 samples from GSM269812 to GSM269833, including the wild type (WT), two independent transgenic lines (T6 and T8), and the vector control line (C2), which was used as an additional control. In all related experiments, the relative water content (RWC) of wild type and transgenic leaves during a period of dehydration was monitored. At day 7 when the transgenic plants were still at an RWC of >85%, the wild type and vector control plants were at an RWC of ∼50–60% [17]. Hence, we first chose experimental samples just on T6 and T8, which were expected to reveal drought tolerant genes. Table 1 gives the specific description of the data used, and the class label (0/1) is according to the different stress conditions. Table 2 presents the susceptibility genotype (WT and C2) data, which were used to discard the tuning genes as described later.

**Table 1:** Resistant samples description.

N.	Sample	Transgenic line	Stress condition	Rep*	Class label+
1	GSM269814	T6	well watered	1	1
2	GSM269815	T6	drought	1	0
3	GSM269816	T8	well watered	1	1
4	GSM269817	T8	drought	1	0
5	GSM269822	T6	well watered	2	1
6	GSM269823	T6	drought	2	0
7	GSM269824	T8	well watered	2	1
8	GSM269825	T8	drought	2	0
9	GSM269830	T6	well watered	3	1
10	GSM269831	T6	drought	3	0
11	GSM269832	T8	well watered	3	1
12	GSM269833	T8	drought	3	0

*: Rep is the number of biological replications.

+ : Class label is used to indicate well watered (1) and the drought (0), respectively.

**Table 2:** Susceptible samples description.

N.	Sample	Genotype	Stress condition	Rep	Class label
1	GSM269812	WT	well watered	1	1
2	GSM269813	WT	drought	1	0
3	GSM269818	C2	well watered	1	1
4	GSM269819	C2	drought	1	0
5	GSM269820	WT	well watered	2	1
6	GSM269821	WT	drought	2	0
7	GSM269826	C2	well watered	2	1
8	GSM269827	C2	drought	2	0
9	GSM269828	WT	well watered	3	1
10	GSM269829	WT	drought	3	0

Table caption follows Table 1.

### Data preprocessing stage

We applied a quantile-based [18] RMA (Robust Multi-chip Averages) method for normalizing microarray data. The RMA feeds probes data stored in Affymetrix CEL into a stochastic model to estimate gene expression and converts the probe data to gene expression data. We conducted the RMA analysis using Bioconductor (http://www.bioconductor.org/), which is an open-source tool for bioinformatics using the R statistical programming language.

### T-test method for preliminary selection

For tens of thousands of genes, it would be of high complexity to use the SVM-RFE directly. Hence, we first employed a t-test [19] to filter out unlikely genes involved in drought tolerance. In our preliminary selection we assigned 0.001 as the p-value threshold, resulting in 736 genes, which are still too many for agronomic studies.

### Using SVM-RFE method for gene selection

RFE is an iterative procedure for SVM classifier. A cost function 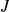 computed on training samples is used as an objective function. Expanding 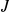 in Taylor series to the second order using the OBD algorithm [20], and neglecting the first order term at the optimum of 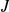, yield:
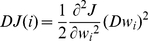
(1)


Here 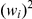 was used as the ranking criterion and we used LIBSVM (a library for Support Vector Machines) [21] with a linear kernel. We present below an outline of the SVM-RFE in the linear kernel. For more details about this method, see Guyon *et al.* [7].

**Algorithm SVM-RFE.**

Inputs:

Training samples (microarray datasets)
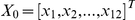
Class labels (1 for well watered or 0 for drought)
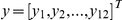



Initialize:

Surviving genes
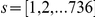
Gene-ranking list
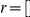
Limit training samples to good genes
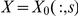
Train the classifier
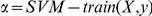
Compute the weight from each selected gene:
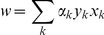
where k indicates the k-th training pattern.

Compute the ranking criterion for the i-th gene
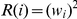
Mark the gene with the lowest ranking
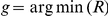
Renew the gene-ranking list
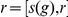
Eliminate the gene with the lowest ranking
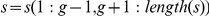
Repeat until 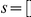


Output:

A gene-ranking list 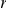.

We trained the classifier, computed the ranking for the 736 genes obtained and then removed the gene with the lowest ranking. We repeated the process until all the genes were removed. This iterative process is a sequence backward selection (SBS) procedure and at last the method produces a gene-ranking list with weights from high to low.

**Figure 1:**
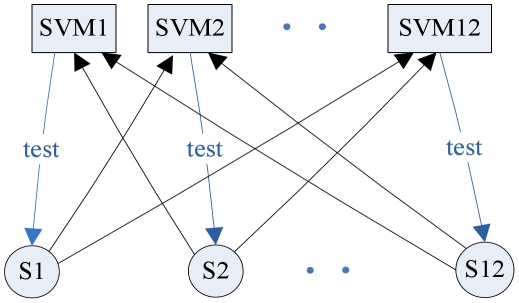
LOOCV for twelve samples.

To improve prediction accuracy we conducted several rounds of bootstrapping in the SVM-RFE procedure and in each round one sorted list was produced. However, there is a shortage of experimental samples that are needed to train the SVM. This brings up two issues: one is how to generate training and test sets, and the other is how to combine the weights of each gene in each sorted list. To address these two issues, we developed the following two solutions:

1. In order to make good use of limited data for predicting drought-resistant genes in *Arabidopsis*, the generation of training set is a key factor. The dataset was randomly split into n subsets of approximately equal size, then one subset was removed, and the remaining samples formed the training set. Each time a different subset was selected in such a way that all the samples had an equal chance to be selected as the training data. We call it n-CV, where n could be equal to 12, 6, 4, or 3, respectively. In the following examples, there are 12 samples from transgenic lines described in Table 1, and a 6-CV was used for each subset with two samples. Then the Leave One Out Cross Validation (LOOCV) was used for training SVM (see Figure 1). After that we performed 100 times of 6-CV and each 6-CV ran 6 times of the SVM-RFE procedure. The computational complexity of each n-CV is n*(g*O(s^3^)), where s is the number of samples and g is the number of genes [22]. The computational time on the training stage primarily depends on the number of genes given the small number of training samples. Hence, the computational complexity can be easily handled by filtering out unlikely genes involved. The 100 times 6-CV training took around 90 minutes to get the results on a desktop computer with Intel Core 2 Duo E6750 and DDR2 2GB memory.
2. We acquired 100 sorted lists, and designed a re-ranking measure to take occurrence and ranking of every gene into account to form a final ranking list of all genes:
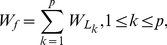
(2)where p is the number of times for CV, 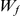 is the sum of one gene's weights in p experiments, and 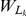 is the weight in the k-th occurrence in sorted lists. 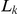 shows the ranking of one feature in the k-th list.
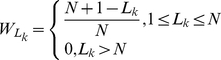
(3)

**Figure 2:**
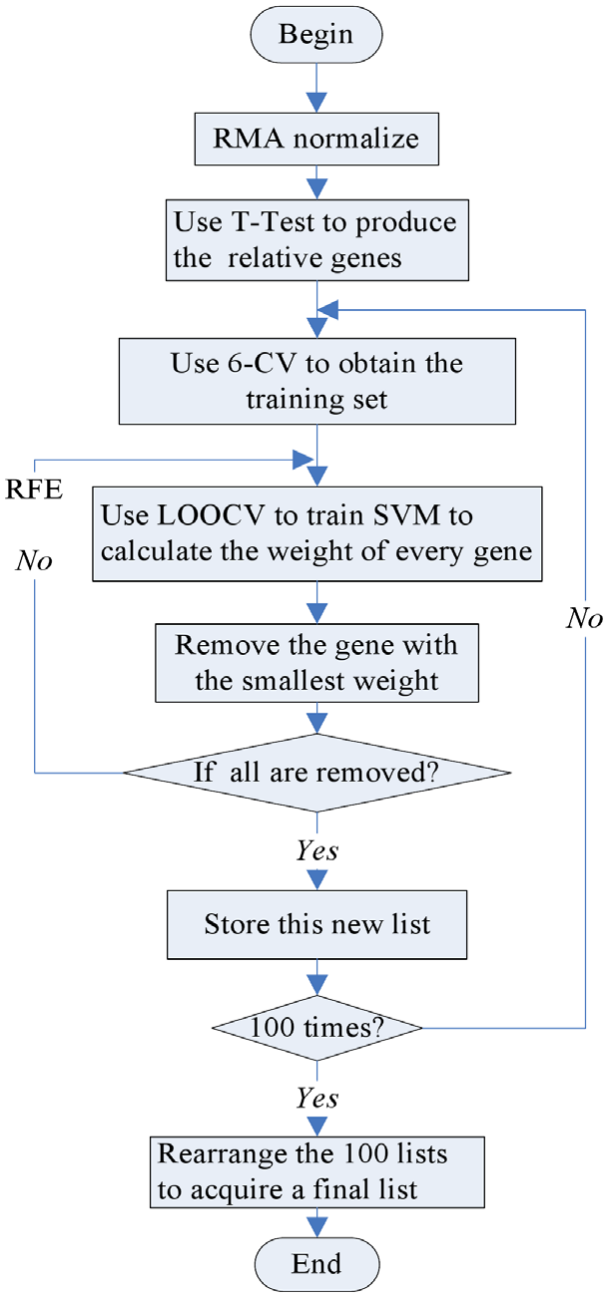
Algorithm flowchart for identifying drought-resistant genes.

Here, we have top 10 (*N*) genes and 100 (*p*) SVM-RFE trainings. Thus, we obtain a final list containing the optimal genes sorted by 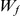 in decreasing order. The algorithm flowchart is shown in Figure 2.

## Results and Discussion

**Figure 3:**
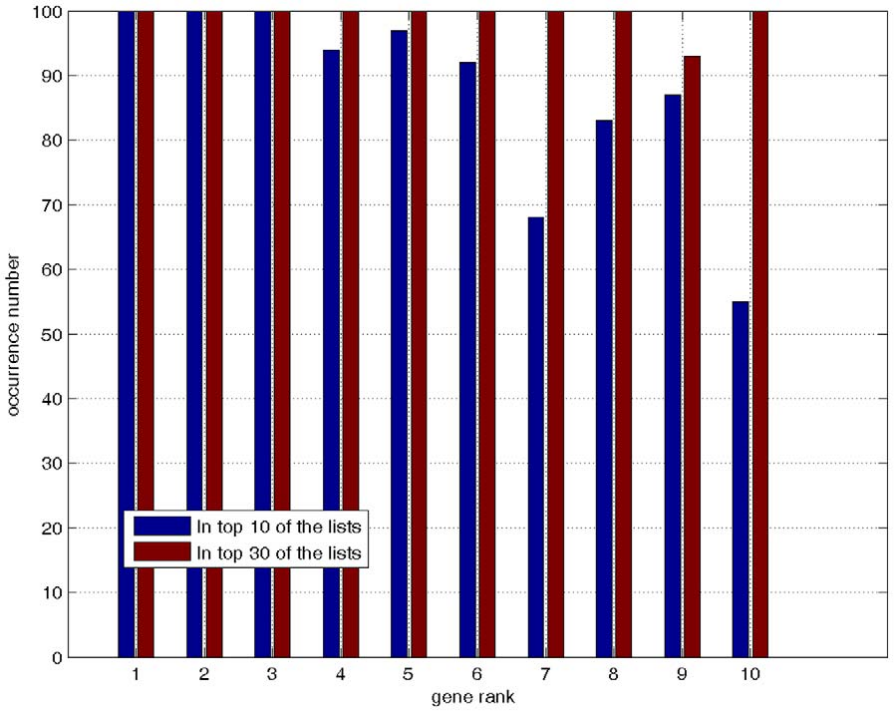
The occurrence of selected 10 genes in the top-10 and top-30 lists when conducting 100 times of 6-CV. The gene order is the same as that in Table 3.

By applying the SVM-RFE method in analyzing the drought-resistant genotype based on the microarray data, we selected 10 genes in the final list according to 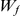 in Eq. 2, as summarized in Table 3. Figure 3 shows the occurrence of these genes in top-10 list and also in top-30 list when conducting 100 times of 6-CV. It shows that the occurrence of these genes in the top list is very high and consistent. We checked the functional annotations using GO (http://www.geneontology.org/), KEGG pathways (http://www.genome.jp/kegg/) and the literatures [2], [23]–[28], and obtained the related information shown in Table 3. From Table 3 it can be seen that most of the genes have no specific molecular function annotations. This is not surprising as the water stress tolerance is a complex trait, which resulted from sophisticated coordination of physiological and biochemical alterations at the cellular and molecular levels [24].

**Table 3:** Selected 10 genes related to drought-resistant genotype.

Rank	Probe ID	Platform ORF	Gene Title	GO: Function	GO: Process	GO: Component
1	248352_at	At5g52300	LTI65(LOW-TEMPERATURE-INDUCED 65)		abscisic acid mediated signaling pathway/response to abscisic acid stimulus/response to cold/response to salt stress/response to water deprivation	
2	247723_at	At5g59220	Protein phosphatase 2C,putative/PP2C, putative	catalytic activity/protein serine/hreonine phosphatase activity	response to abscisic acid stimulus/ response to water deprivation	chloroplast
3	249052_at	At5g44420	PDF1.2		defense response	cell wall / endomembrane system
4	265342_at	At2g18300	basic helix-loop-helix (bHLH) family protein			nucleus
5	257365_x_at	At2g26020	PDF1.2b(plant defensin 1.2b)		defense response	cell wall / endomembrane system
6	266743_at	At2g02990	RNS1(RIBONUCLEASE1); endoribonuclease/ribonuclease	endoribonuclease activity/ribonuclease activity	response to wounding	cell wall/extracellular region/plasma membrane
7	258897_at	At3g05730	hypothetical protein			endomembrane system
8	266462_at	At2g47770	benzodiazepine receptor-related		response to abscisic acid stimulus/response to osmotic stress/response to salt stress	Golgi stack/endoplasmic reticulum/membrane
9	248218_at	At5g53710	hypothetical protein			
10	262347_at	At1g64110	AAA-type ATPase family protein	ATP binding/nucleotide binding		

Table 3 reveals some interesting biological features. Both the first and second genes directly respond to water deprivation. The cellular component of the second gene is chloroplast where photosynthesis of plants occurs. As photosynthesis uses carbon dioxide and water releasing oxygen, it is likely that this gene plays some role in water utilization in chloroplast. The third and fifth genes are both related to defense response, whose pathway is known to have cross-talk with drought resistance pathway [29].

The sixth gene in the table responds to wounding and it may help repair the damage of cell caused by water loss. Some other genes in the list may also be related to drought resistance. The eighth-ranked gene responds to osmotic stress and salt stress. Osmotic adjustment is an important physiological mechanism adapting to water stress [24]. Osmotic adjustment can maintain a dynamic balance between damage and repair of cellular components to relieve plants injury and improve plants' ability of stress resistance.

In the column GO: Component of Table 3, all of the 3^rd^, 5^th^, 6^th^, 7^th^, and 8^th^ genes belong to membrane systems, like endomembrane, plasma membrane, and so on. Membrane system is the key part damaged by drought stress and it is the most sensitive original reaction site against adversity [25]. Membrane, together with associated proteins, provides cells with not only a relatively stable internal environment, but also provides a switch to material transportation, energy exchange and information transmission between cells and the environment. Therefore, these five genes may help adjust osmotic membranes to boost drought resistance. In all we have demonstrated that seven genes may be closely related to water tolerance for *Arabidopsis*, i.e., the 1^st^, 2^nd^, 3^rd^, 5^th^, 6^th^, 7^th^, and 8^th^ genes in Table 3.

We also repeated the computational process with the susceptibility genotype samples (WT and C2), and obtained the top-10 gene list as described in Table 4. From the relative function annotations, it appears that most of these genes have little relationship with the ability of drought resistance, which may explain why these genotypes do not have capacities of drought resistance.

**Table 4:** Selected 10 related to water-susceptibility genotype.

Rank	Probe ID	Platform ORF	Gene Title	GO: Function	GO: Process	GO: Component
1	262128_at	At1g52690	late embryogenesis abundant protein, putative / LEA protein, putative		embryonic development ending in seed dormancy	
2	264580_at	At1g05340	hypothetical protein		biological_process	
3	258499_at	At3g02540	RAD23-3 (PUTATIVE DNA REPAIR PROTEIN RAD23-3); damaged DNA binding	proteasome binding///ubiquitin binding	nucleotide-excision repair/proteasomal ubiquitin-dependent protein catabolic process	nucleus
4	258239_at	At3g27690	LHCB2.3; chlorophyll binding	chlorophyll binding	photosynthesis/response to blue light/response to far red light/response to red light	chloroplast envelope/chloroplast thylakoid membrane/light-harvesting complex/thylakoid
5	266462_at	At2g47770	benzodiazepine receptor-related		response to abscisic acid stimulus/response to osmotic stress/response to salt stress	Golgi stack/endoplasmic reticulum/membrane
6	258347_at	At3g17520	late embryogenesis abundant domain-containing protein / LEA domain-containing protein		embryonic development ending in seed dormancy	
7	247095_at	At5g66400	RAB18 (RESPONSIVE TO ABA 18)		cold acclimation/response to 1-aminocyclopropane-1-carboxylic acid/response to abscisic acid stimulus/response to stress/response to water deprivation	
8	247723_at	At5g59220	protein phosphatase 2C, putative / PP2C, putative	catalytic activity/protein serine/threonine phosphatase activity	response to abscisic acid stimulus/response to water deprivation	chloroplast
9	262382_at	At1g72920	disease resistance protein (TIR-NBS class), putative	transmembrane receptor activity		intrinsic to membrane
10	248352_at	At5g52300	LTI65 (LOW-TEMPERATURE-INDUCED 65)		abscisic acid mediated signaling pathway/response to abscisic acid stimulus/response to cold/response to salt stress/response to water deprivation	

Our understanding of the functions of these seven genes is far from complete. Compared the top-10 gene list obtained from the resistant genotype with the susceptibility genotype list, 3 genes are the same, which are the 1^st^, 2^nd^, and 8^th^ in Table 3, and the 10^th^, 8^th^, and 5^th^ in Table 4. We call the same ones tuning genes [16]. Maybe their adaptability to hydropenia is the result of irritable reactions to environmental changes. So by removing the tuning genes from the top-10 gene list with transgenic genotype, the inference is that the real drought-resistant genes should be in the 7 ones in Table 5 (a subset of Table 3). And with the same thought, we analyzed the two top 100 gene lists using the resistant genotype and the susceptibility genotype, respectively (see Table S1 and Table S2 for the detailed information). Comparing the top-100 gene list obtained from the resistant genotype with the susceptibility genotype list, it can be seen that 37 genes overlap (tuning genes). In Table S1, the “Overlap” column indicates the tuning genes. We compared our result against the result published by Huang *et al.* [30] to look for the overlap of the genes identified to be involved in drought resistance. Our comparison shows that 5 out of the 10 genes from resistant genotype and 6 out of the 10 genes from susceptible genotype are identified to be the same. When we expanded our analysis to include the top 100 genes, 50 genes of the resistant genotype and 42 of the susceptible genotype overlap with the gene lists in the published results. GO term enrichment analysis was also performed for the annotations of the top 100 genes in the resistant and susceptibility genotypes, respectively using Amigo GO Term Enrichment Tool (http://amigo.geneontology.org/cgi-bin/amigo/term_enrichment) as shown in Table 6. All the GO terms identified for both genotypes with a significant p-value less than 0.001 have functional categories related to the drought stress.

**Table 5:** The new list with the tuning genes removed from the top-10 resistant gene list.

Rank	1	2	3	4	5	6	7
**Probe ID**	249052_at	265342_at	257365_x_at	266743_at	258897_at	248218_at	262347_at
**Platform ORF**	At5g44420	At2g18300	At2g26020	At2g02990	At3g05730	At5g53710	At1g64110

**Table 6:** GO Term Enrichment for resistant and susceptibility genotypes.

Resistant Genotype	GO Term	Aspect	P-value	Sample frequency	Background frequency
	GO:0050896 response to stimulus	P	1.49e-04	36/99 (36.4%)	4570/29887 (15.3%)
	GO:0006950 response to stress	P	5.10e-04	23/99 (23.2%)	2221/29887 (7.4%)
	GO:0009628 response to abiotic stimulus	P	6.17e-04	18/99 (18.2%)	1421/29887 (4.8%)
	GO:0009725 response to hormone stimulus	P	1.74e-03	14/99 (14.1%)	935/29887 (3.1%)
	GO:0009719 response to endogenous stimulus	P	4.41e-03	14/99 (14.1%)	1014/29887 (3.4%)
	GO:0009611 response to wounding	P	7.93e-03	6/99 (6.1%)	151/29887 (0.5%)
	GO:0042221 response to chemical stimulus	P	9.68e-03	20/99 (20.2%)	2085/29887 (7.0%)
**Susceptibility Genotype**	GO:0009628 response to abiotic stimulus	P	5.47e-04	18/100 (18.0%)	1421/29887 (4.8%)
	GO:0009266 response to temperature stimulus	P	4.96e-03	9/100 (9.0%)	407/29887 (1.4%)

The SVM-RFE algorithm was used in cancer marker gene prediction with abundant datasets. However, there are much fewer microarray samples for finding the drought-resistant genes of *Arabidopsis*. Hence, we modified the original SVM-RFE method to address this drawback by using bootstrapping and leave-one-out cross-validation. Our study shows that the improved method is effective for identifying drought-resistance genes. Since the sparseness of gene expression data for studying genotype-trait relationships is a common issue, the method provides a framework for handling this issue. The framework has some advantages over some other feature selection methods that require extensive training data, such as random forest [31]. This is by no means a replacement of additional experimental data, but it can effectively utilize the sparse data available to generate useful hypotheses and guide further targeted experimental work.

Although *Arabidopsis* is a model organism for plant gene function analysis and gene expression studies, few genes related to drought resistance mechanisms are annotated with direct experimental evidences. There is a need to predict additional drought-resistance genes based on gene expression data. The predictions of drought-resistance genes generated by the JU/MU development of the SVM-RFE method-based software provides useful hypothesis for experimentalists to verify. For example, the 3^rd^, 8^th^ and 10^th^ genes in Table 3 are not annotated as drought-resistance genes, but they are highly likely involved in drought resistance. Perhaps a major challenge in the future is to inquire into the relative contribution of each gene to water tolerance. The JU/MU approach is applicable to the study of plant genes related to other stress resistance and genes associated with any agronomic trait in general.

## Supporting Information

Table S1 Detailed information of top 100 genes from resistant genotype.
(DOC)

Table S2 Detailed information of top 100 genes from susceptibility genotype.
(DOC)
