# Supplementary material for: Prediction of Drought-Resistant Genes in Arabidopsis thaliana Using SVM-RFE
Source: PLoS One. 2011 Jul 15;6(7):e21750. doi: 10.1371/journal.pone.0021750 (PMC3137602; doi:10.1371/journal.pone.0021750)
Supplement: Table S1 — Detailed information of top 100 genes from resistant genotype. (DOC) [file pone.0021750.s001.doc]

**Table S1. Detailed information of top 100 genes from resistant genotype**

| **Rank** | **Overlap** | **Probe ID** | **ORF** | **Gene Title** | **Gene Symbol** | **GO: Function** | **GO: Process** | **GO: Component** |
| --- | --- | --- | --- | --- | --- | --- | --- | --- |
| 1 | 1 | 248352_at | At5g52300 | LTI65 (LOW-TEMPERATURE-INDUCED 65) | LTI65 |  | abscisic acid mediated signaling pathway///response to abscisic acid stimulus///response to cold///response to salt stress///response to water deprivation |  |
| 2 | 1 | 247723_at | At5g59220 | protein phosphatase 2C, putative / PP2C, putative | AT5G59220 | catalytic activity///protein serine/threonine phosphatase activity | response to abscisic acid stimulus///response to water deprivation | chloroplast |
| 3 | 1 | 249052_at | At5g44420 | PDF1.2 | PDF1.2 |  | defense response/// jasmonic acid and ethylene-dependent systemic resistance///response to ethylene stimulus///response to insect///response to jasmonic acid stimulus///NOT response to salicylic acid stimulus | cell wall/// endomembrane system |
| 4 | 1 | 265342_at | At2g18300 | basic helix-loop-helix (bHLH) family protein | AT2G18300 |  |  | nucleus |
| 5 | 0 | 257365_x_at | At2g26020 | PDF1.2b (plant defensin 1.2b) | PDF1.2b |  | defense response | cell wall///endomembrane system |
| 6 | 0 | 266743_at | At2g02990 | RNS1 (RIBONUCLEASE 1); endoribonuclease/ ribonuclease | RNS1 | endoribonuclease activity///ribonuclease activity | aging///anthocyanin biosynthetic process///cellular response to phosphate starvation///NOT jasmonic acid mediated signaling pathway///response to wounding | cell wall/// extracellular region///plasma membrane |
| 7 | 0 | 258897_at | At3g05730 | hypothetical protein | AT3G05730 |  |  | endomembrane system |
| 8 | 1 | 266462_at | At2g47770 | benzodiazepine receptor-related | AT2G47770 |  | response to abscisic acid stimulus///response to osmotic stress///response to salt stress | Golgi stack///endoplasmic reticulum///membrane |
| 9 | 0 | 248218_at | At5g53710 | hypothetical protein | AT5G53710 |  |  |  |
| 10 | 1 | 262347_at | At1g64110 | AAA-type ATPase family protein | AT1G64110 | ATP binding///nucleotide binding |  |  |
| 11 | 0 | 264262_at | At1g09200 | histone H3 | AT1G09200 | DNA binding | nucleosome assembly | chloroplast///nucleosome///nucleus |
| 12 | 0 | 246275_at | At4g36540 | BEE2 (BR Enhanced Expression 2); DNA binding / transcription factor | BEE2 | DNA binding///transcription factor activity | regulation of transcription | nucleus |
| 13 | 1 | 261957_at | At1g64660 | ATMGL (ARABIDOPSIS THALIANA METHIONINE GAMMA-LYASE); catalytic/ methionine gamma-lyase | ATMGL | catalytic activity///methionine gamma-lyase activity | cellular amino acid metabolic process///methionine catabolic process via 2-oxobutanoate | cytosol |
| 14 | 1 | 266503_at | At2g47780 | rubber elongation factor (REF) protein-related | AT2G47780 |  |  |  |
| 15 | 1 | 247377_at | At5g63180 | pectate lyase family protein | AT5G63180 | lyase activity///pectate lyase activity |  |  |
| 16 | 0 | 266327_at | At2g46680 | ATHB-7 (ARABIDOPSIS THALIANA HOMEOBOX 7); transcription activator/ transcription factor | ATHB-7 |  | transcription activator activity///transcription factor activity | regulation of transcription, DNA-dependent///response to abscisic acid stimulus///response to water deprivation |
| 17 | 1 | 257206_at | At3g16530 | legume lectin family protein | AT3G16530 | sugar binding |  | apoplast///cell wall///nucleus///plant-type cell wall |
| 18 | 1 | 248505_at | At5g50360 | hypothetical protein | AT5G50360 |  |  |  |
| 19 | 0 | 250500_at | At5g09530 | hydroxyproline-rich glycoprotein family protein | AT5G09530 |  |  |  |
| 20 | 0 | 256789_at | At3g13672 | seven in absentia (SINA) family protein | AT3G13672 |  | multicellular organismal development///ubiquitin-dependent protein catabolic process | nucleus |
| 21 | 1 | 258100_at | At3g23550 | MATE efflux family protein | AT3G23550 | antiporter activity///transporter activity |  | membrane |
| 22 | 1 | 266098_at | At2g37870 | protease inhibitor/seed storage/lipid transfer protein (LTP) family protein | AT2G37870 | lipid binding | lipid transport | endomembrane system |
| 23 | 1 | 249894_at | At5g22580 | hypothetical protein | AT5G22580 |  |  |  |
| 24 | 0 | 265400_at | At2g10940 | protease inhibitor/seed storage/lipid transfer protein (LTP) family protein | AT2G10940 |  | lipid transport |  |
| 25 | 0 | 263881_at | At2g21820 | hypothetical protein | AT2G21820 |  |  |  |
| 26 | 0 | 259479_at | At1g19020 | hypothetical protein | AT1G19020 |  | response to oxidative stress |  |
| 27 | 1 | 260357_at | At1g69260 | AFP1 (ABI FIVE BINDING PROTEIN) | AFP1 | abscisic acid mediated signaling pathway | nucleus |  |
| 28 | 0 | 247651_at | At5g59870 | HTA6; DNA binding | HTA6 | DNA binding///DNA binding | nucleosome assembly | nucleolus |
| 29 | 0 | 264318_at | At1g04220 | KCS2 (3-KETOACYL-COA SYNTHASE 2); fatty acid elongase | KCS2 | fatty acid elongase activity | response to osmotic stress///response to wounding///suberin biosynthetic process | membrane |
| 30 | 1 | 247095_at | At5g66400 | RAB18 (RESPONSIVE TO ABA 18) | RAB18 |  | cold acclimation///response to 1-aminocyclopropane-1-carboxylic acid///response to abscisic acid stimulus///response to stress///response to water deprivation |  |
| 31 | 0 | 264672_at | At1g09750 | chloroplast nucleoid DNA-binding protein-related | AT1G09750 | aspartic-type endopeptidase activity | proteolysis | apoplast///cell wall///plant-type cell wall |
| 32 | 0 | 258809_at | At3g04070 | anac047(Arabidopsis NAC domain containing protein 47); transcription factor | anac047 | transcription factor activity | multicellular organismal development///regulation of transcription |  |
| 33 | 1 | 248236_at | At5g53870 | plastocyanin-like domain-containing protein | AT5G53870 | copper ion binding/// copper ion binding/// electron carrier activity |  | anchored to membrane///plasma membrane |
| 34 | 0 | 266790_at | At2g28950 | ATEXPA6 (ARABIDOPSIS THALIANA EXPANSIN A6) | ATEXPA6 |  | plant-type cell wall loosening///plant-type cell wall loosening///plant-type cell wall modification involved in multidimensional cell growth///syncytium formation///unidimensional cell growth | plant-type cell wall |
| 35 | 0 | 264240_at | At1g54820 | protein kinase family protein | AT1G54820 | ATP binding ///protein serine/threonine kinase activity///protein tyrosine kinase activity | protein amino acid phosphorylation |  |
| 36 | 0 | 259442_at | At1g02310 | glycosyl hydrolase family protein 5 / cellulase family protein / (1-4)-beta-mannan endohydrolase, putative | AT1G02310 | catalytic activity///cation binding///hydrolase activity, hydrolyzing O-glycosyl compounds | carbohydrate metabolic process | endomembrane system |
| 37 | 1 | 246487_at | At5g16030 | hypothetical protein | AT5G16030 |  |  |  |
| 38 | 1 | 250648_at | At5g06760 | late embryogenesis abundant group 1 domain-containing protein / LEA group 1 domain-containing protein | AT5G06760 |  | embryonic development ending in seed dormancy |  |
| 39 | 1 | 254250_at | At4g23290 | protein kinase family protein | AT4G23290 | kinase activity | protein amino acid phosphorylation | mitochondrion |
| 40 | 1 | 267080_at | At2g41190 | amino acid transporter family protein | AT2G41190 | amino acid transmembrane transporter activity | amino acid transport | membrane |
| 41 | 0 | 260783_at | At1g06160 | ORA59 (OCTADECANOID-RESPONSIVE ARABIDOPSIS AP2/ERF 59); DNA binding / transcription activator/ transcription factor | ORA59 | DNA binding///transcription activator activity///transcription factor activity///transcription factor activity | ethylene mediated signaling pathway///jasmonic acid and ethylene-dependent systemic resistance///regulation of transcription, DNA-dependent///response to ethylene stimulus///response to jasmonic acid stimulus |  |
| 42 | 0 | 260097_at | At1g73220 | AtOCT1 (Arabidopsis thaliana ORGANIC CATION/CARNITINE TRANSPORTER1); carbohydrate transmembrane transporter/ carnitine transporter/ transporter | AtOCT1 | carbohydrate transmembrane transporter activity///carnitine transporter activity///transporter activity |  | membrane///plasma membrane |
| 43 | 0 | 260005_at | At1g67920 | hypothetical protein | AT1G67920 |  |  |  |
| 44 | 0 | 256603_at | At3g28270 | hypothetical protein | AT3G28270 |  |  |  |
| 45 | 0 | 259632_at | At1g56430 | NAS4 (NICOTIANAMINE SYNTHASE 4); nicotianamine synthase | NAS4 | nicotianamine synthase activity | nicotianamine biosynthetic process |  |
| 46 | 1 | 263544_at | At2g21590 | APL4; glucose-1-phosphate adenylyltransferase | APL4 | glucose-1-phosphate adenylyltransferase activity///glucose-1-phosphate adenylyltransferase activity | starch biosynthetic process | glucose-1-phosphate adenylyltransferase complex |
| 47 | 1 | 262382_at | At1g72920 | disease resistance protein (TIR-NBS class), putative | AT1G72920 | transmembrane receptor activity |  | intrinsic to membrane |
| 48 | 0 | 253519_at | At4g31240 | hypothetical protein | AT4G31240 |  |  |  |
| 49 | 0 | 260831_at | At1g06830 | glutaredoxin family protein | AT1G06830 | electron carrier activity///protein disulfide oxidoreductase activity | cell redox homeostasis | endomembrane system |
| 50 | 0 | 255527_at | At4g02360 | hypothetical protein | AT4G02360 |  |  |  |
| 51 | 1 | 264195_at | At1g22690 | gibberellin-responsive protein, putative | AT1G22690 |  | response to gibberellin stimulus |  |
| 52 | 1 | 251137_at | At5g01300 | phosphatidylethanolamine-binding family protein | AT5G01300 | phosphatidylethanolamine binding |  |  |
| 53 | 0 | 251775_s_at | At3g55610 | P5CS2 (DELTA 1-PYRROLINE-5-CARBOXYLATE SYNTHASE 2); catalytic/ glutamate 5-kinase/ glutamate-5-semialdehyde dehydrogenase/ oxidoreductase///P5CS1 (DELTA1-PYRROLINE-5-CARBOXYLATE SYNTHASE 1); delta1-pyrroline-5-carboxylate synthetase | P5CS2///P5CS1 | catalytic activity///glutamate 5-kinase activity///glutamate-5-semialdehyde dehydrogenase activity///oxidoreductase activity///delta1-pyrroline-5-carboxylate synthetase activity | embryonic development ending in seed dormancy///hyperosmotic salinity response///proline biosynthetic process ///response to abscisic acid stimulus///hyperosmotic salinity response///proline biosynthetic process /// response to desiccation///response to oxidative stress///response to salt stress///response to water deprivation///root development | chloroplast/// cytoplasm/// chloroplast// /cytoplasm///membrane |
| 54 | 0 | 250434_at | At5g10390 | histone H3 | AT5G10390 | DNA binding | nucleosome assembly | chloroplast///nucleosome |
| 55 | 0 | 266550_s_at | At2g35070 | hypothetical protein///hypothetical protein | AT2G35090///AT2G35070 |  | ///pollen development///pollen tube development |  |
| 56 | 0 | 264510_at | At1g09530 | PIF3 (PHYTOCHROME INTERACTING FACTOR 3); DNA binding / protein binding / transcription factor/ transcription regulator | PIF3 | DNA binding///DNA binding///protein binding///transcription factor activity///transcription regulator activity///transcription regulator activity | de-etiolation///gibberellic acid mediated signaling pathway///positive regulation of anthocyanin metabolic process///red or far-red light signaling pathway///response to red or far red light///signal transduction | nucleus |
| 57 | 0 | 258091_at | At3g14560 | hypothetical protein | AT3G14560 |  |  |  |
| 58 | 0 | 259133_at | At3g05400 | sugar transporter, putative | AT3G05400 | carbohydrate transmembrane transporter activity |  | membrane |
| 59 | 0 | 248763_at | At5g47550 | cysteine protease inhibitor, putative / cystatin, putative | AT5G47550 | cysteine-type endopeptidase inhibitor activity |  | cell wall |
| 60 | 0 | 253407_at | At4g32920 | glycine-rich protein | AT4G32920 | molecular_function |  |  |
| 61 | 0 | 249467_at | At5g39610 | ATNAC6 (ARABIDOPSIS NAC DOMAIN CONTAINING PROTEIN 6); protein heterodimerization/ transcription factor | ATNAC6 | NOT protein heterodimerization activity///protein homodimerization activity///transcription factor activity///transcription factor activity | leaf senescence///leaf senescence///multicellular organismal development///regulation of gene expression///response to oxidative stress///response to salt stress///senescence | nucleus |
| 62 | 0 | 257474_at | At1g80850 | methyladenine glycosylase family protein | AT1G80850 | DNA-3-methyladenine glycosylase I activity///catalytic activity | DNA repair///base-excision repair |  |
| 63 | 0 | 251154_at | At3g63110 | ATIPT3 (ARABIDOPSIS THALIANA ISOPENTENYLTRANSFERASE 3); ATP binding /tRNA isopentenyltransferase/transferase, transferring alkyl or aryl(other than methyl) groups | ATIPT3 | adenylate dimethylallyltransferase activity///transferase activity, transferring alkyl or aryl (other than methyl) groups | cytokinin biosynthetic process///cytokinin biosynthetic process | nucleus///plastid |
| 64 | 0 | 251759_at | At3g55630 | ATDFD (A. THALIANA DHFS-FPGS HOMOLOG D); tetrahydrofolylpolyglutamate synthase | ATDFD | tetrahydrofolylpolyglutamate synthase activity | one-carbon metabolic process | cytosol |
| 65 | 0 | 246596_at | At5g14740 | CA2 (CARBONIC ANHYDRASE 2); carbonate dehydratase/ zinc ion binding | CA2 | carbonate dehydratase activity///zinc ion binding | carbon utilization///defense response to bacterium | chloroplast///chloroplast stroma///chloroplast thylakoid membrane |
| 66 | 0 | 266865_at | At2g29980 | FAD3 (FATTY ACID DESATURASE 3); omega-3 fatty acid desaturase | FAD3 | omega-3 fatty acid desaturase activity/omega-3 fatty acid desaturase activity | unsaturated fatty acid biosynthetic process | endoplasmic reticulum |
| 67 | 1 | 256114_at | At1g16850 | hypothetical protein | AT1G16850 |  | response to salt stress |  |
| 68 | 0 | 251524_at | At3g58990 | aconitase C-terminal domain-containing protein | AT3G58990 | 3-isopropylmalate dehydratase activity///hydro-lyase activity | leucine biosynthetic process///metabolic process | plastid |
| 69 | 1 | 259161_at | At3g01500 | CA1 (CARBONIC ANHYDRASE 1); carbonate dehydratase/ zinc ion binding | CA1 | carbonate dehydratase activity///carbonate dehydratase activity///zinc ion binding///zinc ion binding | carbon utilization ///defense response to fungus, incompatible interaction///regulation of stomatal movement ///response to carbon dioxide///response to cold | apoplast///chloroplast///chloroplast thylakoid membrane///membrane///stromule///thylakoid |
| 70 | 0 | 265722_at | At2g40100 | LHCB4.3 (light harvesting complex PSII); chlorophyll binding | LHCB4.3 | chlorophyll binding | photosynthesis///response to blue light///response to far red light///response to red light | chloroplast thylakoid membrane///light-harvesting complex///membrane |
| 71 | 0 | 256825_at | At3g22120 | CWLP (CELL WALL-PLASMA MEMBRANE LINKER PROTEIN); lipid binding | CWLP | lipid binding | lipid transport///lipid transport | endomembrane system |
| 72 | 0 | 262507_at | At1g11330 | S-locus lectin protein kinase family protein | AT1G11330 | ATP binding///protein kinase activity///protein serine/threonine kinase activity///protein tyrosine kinase activity///sugar binding | protein amino acid phosphorylation///recognition of pollen | plasma membrane |
| 73 | 1 | 250942_at | At5g03350 | legume lectin family protein | AT5G03350 | sugar binding |  | apoplast///cell wall///chloroplast |
| 74 | 0 | 251766_at | At3g55910 | hypothetical protein | AT3G55910 |  |  |  |
| 75 | 1 | 253373_at | At4g33150 | lysine-ketoglutarate reductase/saccharopine dehydrogenase bifunctional enzyme | AT4G33150 | saccharopine dehydrogenase activity | L-lysine catabolic process | cytoplasm |
| 76 | 0 | 255822_at | At2g40610 | ATEXPA8 (ARABIDOPSIS THALIANA EXPANSIN A8) | ATEXPA8 |  | plant-type cell wall loosening/// plant-type cell wall loosening/// plant-type cell wall modification involved in multidimensional cell growth/// syncytium formation/// unidimensional cell growth/// unidimensional cell growth | endomembrane system///extracellular region |
| 77 | 0 | 258158_at | At3g17790 | PAP17; acid phosphatase/phosphatase/protein serine/threonine phosphatase | PAP17 | acid phosphatase activity///phosphatase activity///protein serine/threonine phosphatase activity | cellular phosphate ion homeostasis///response to hydrogen peroxide | cell surface |
| 78 | 0 | 248756_at | At5g47560 | TDT (TONOPLAST DICARBOXYLATE TRANSPORTER); malate transmembrane transporter/ sodium:dicarboxylate symporter | TDT | malate transmembrane transporter activity///malate transmembrane transporter activity///sodium:dicarboxylate symporter activity | dicarboxylic acid transport///malate transport///regulation of intracellular pH///sodium ion transport | vacuole |
| 79 | 0 | 259922_at | At1g72770 | HAB1 (HOMOLOGY TO ABI1); catalytic/ protein serine/threonine phosphatase | HAB1 | protein serine/threonine phosphatase activity/protein serine/threonine phosphatase activity | protein amino acid dephosphorylation | protein serine/threonine phosphatase complex |
| 80 | 0 | 261913_at | At1g65860 | FMO GS-OX1 (FLAVIN-MONOOXYGENASE GLUCOSINOLATE S-OXYGENASE 1); 3-methylthiopropyl glucosinolate S-oxygenase/ 4-methylthiopropyl glucosinolate S-oxygenase/ 5-methylthiopropyl glucosinolate S-oxygenase/ 6-methylthiopropyl glucosinolate S-oxygenase/ 7-methylthiopropyl glucosinolate S-oxygenase | FMO_GS-OX1 | 3-methylthiopropyl glucosinolate S-oxygenase activity/4-methylthiopropyl glucosinolate S-oxygenase activity/5-methylthiopropyl glucosinolate S-oxygenase activity/6-methylthiopropyl glucosinolate S-oxygenase activity/7-methylthiopropyl glucosinolate S-oxygenase activity/8-methylthiopropyl glucosinolate S-oxygenase activity /monooxygenase activity | glucosinolate biosynthetic process from homomethionine |  |
| 81 | 1 | 258239_at | At3g27690 | LHCB2.3; chlorophyll binding | LHCB2.3 | chlorophyll binding | photosynthesis///response to blue light///response to far red light///response to red light | chloroplast envelope /chloroplast thylakoid membrane/ light-harvesting complex |
| 82 | 0 | 258920_at | At3g10520 | AHB2 (ARABIDOPSIS HAEMOGLOBIN 2); oxygen transporter | AHB2 | oxygen transporter activity | response to cytokinin stimulus |  |
| 83 | 1 | 259892_at | At1g72610 | GER1 (GERMIN-LIKE PROTEIN 1); oxalate oxidase | GER1 | NOT oxalate oxidase activity |  | extracellular matrix |
| 84 | 0 | 247685_at | At5g59680 | leucine-rich repeat protein kinase, putative | AT5G59680 |  | protein amino acid phosphorylation | endomembrane system |
| 85 | 1 | 258139_at | At3g24520 | AT-HSFC1; DNA binding / transcription factor | AT-HSFC1 | DNA binding/ transcription factor activity | regulation of transcription, DNA-dependent | nucleus |
| 86 | 1 | 257271_at | At3g28007 | nodulin MtN3 family protein | AT3G28007 |  |  | endomembrane system/ integral to membrane |
| 87 | 0 | 250408_at | At5g10930 | CIPK5 (CBL-INTERACTING PROTEIN KINASE 5); ATP binding / kinase/ protein kinase/ protein serine/threonine kinase | CIPK5 | ATP binding/kinase activity/protein kinase activity/ protein serine/ threonine kinase activity | protein amino acid phosphorylation///signal transduction |  |
| 88 | 1 | 251928_at | At3g53980 | protease inhibitor/seed storage/lipid transfer protein (LTP) family protein | AT3G53980 | lipid binding | lipid transport |  |
| 89 | 0 | 252827_at | At4g39950 | CYP79B2; electron carrier/ heme binding / iron ion binding / monooxygenase/ oxygen binding | CYP79B2 | electron carrier activity///heme binding///iron ion binding///monooxygenase activity///oxygen binding | camalexin biosynthetic process/defense response/defense response by callose deposition in cell wall/defense response to bacterium/defense response to oomycetes/indoleacetic acid biosynthetic process/ response to bacterium/tryptophan catabolic process | chloroplast |
| 90 | 0 | 265724_at | At2g32100 | OFP16 (ARABIDOPSIS THALIANA OVATE FAMILY PROTEIN 16) | OFP16 |  |  |  |
| 91 | 0 | 248526_at | At5g50730 | metal ion binding | AT5G50740 | metal ion binding |  |  |
| 92 | 0 | 262098_at | At1g56170 | NF-YC2 (NUCLEAR FACTOR Y, SUBUNIT C2); DNA binding / transcription activator/ transcription factor | NF-YC2 | DNA binding///transcription activator activity///transcription factor activity | positive regulation of gene-specific transcription///regulation of transcription, DNA-dependent | CCAAT-binding factor complex///cytoplasm///nucleus///nucleus |
| 93 | 1 | 249454_at | At5g39520 | hypothetical protein | AT5G39520 | molecular_function |  |  |
| 94 | 0 | 253203_at | At4g34710 | ADC2 (ARGININE DECARBOXYLASE 2); arginine decarboxylase | ADC2 | arginine decarboxylase activity | polyamine biosynthetic process///putrescine biosynthetic process///response to abscisic acid stimulus///response to cold///response to jasmonic acid stimulus///response to osmotic stress///response to oxidative stress///response to salt stress///response to wounding///seed development |  |
| 95 | 0 | 247284_at | At5g64410 | OPT4 (OLIGOPEPTIDE TRANSPORTER 4); oligopeptide transporter | OPT4 | oligopeptide transporter activity///oligopeptide transporter activity | oligopeptide transport | membrane |
| 96 | 0 | 255543_at | At4g01870 | tolB protein-related | AT4G01870 |  |  |  |
| 97 | 1 | 262128_at | At1g52690 | late embryogenesis abundant protein, putative / LEA protein, putative | AT1G52690 |  | embryonic development ending in seed dormancy |  |
| 98 | 0 | 254102_at | At4g25050 | ACP4 (acyl carrier protein 4); acyl carrier | ACP4 | acyl carrier activity | fatty acid biosynthetic process///response to light stimulus | chloroplast///chloroplast envelope///chloroplast stroma |
| 99 | 1 | 259786_at | At1g29660 | GDSL-motif lipase/hydrolase family protein | AT1G29660 | carboxylesterase activity |  |  |
| 100 | 0 | 264873_at | At1g24100 | UGT74B1 (UDP-glucosyl transferase 74B1); UDP-glycosyltransferase/ thiohydroximate beta-D-glucosyltransferase/ transferase, transferring glycosyl groups | UGT74B1 | UDP-glycosyltransferase activity///thiohydroximate beta-D-glucosyltransferase activity///transferase activity, transferring glycosyl groups | defense response by callose deposition in cell wall///defense response to bacterium///glucosinolate biosynthetic process |  |

Overlap is used to indicate the overlapping genes (marked by 1) between the top 100 genes obtained from the resistant genotype and the top 100 genes from the susceptibility genotype. There are 37 overlapping genes (tuning genes) in total.
