# Supplementary material for: Prediction of Drought-Resistant Genes in Arabidopsis thaliana Using SVM-RFE
Source: PLoS One. 2011 Jul 15;6(7):e21750. doi: 10.1371/journal.pone.0021750 (PMC3137602; doi:10.1371/journal.pone.0021750)
Supplement: Table S2 — Detailed information of top 100 genes from susceptibility genotype. (DOC) [file pone.0021750.s002.doc]

**Table S2. Detailed information of top 100 genes from susceptibility genotype**

| **Rank** | **Probe ID** | **ORF** | **Gene Title** | **Gene Symbol** | **GO: Function** | **GO: Process** | **GO: Component** |
| --- | --- | --- | --- | --- | --- | --- | --- |
| 1 | 262128_at | At1g52690 | late embryogenesis abundant protein, putative / LEA protein, putative | AT1G52690 |  | embryonic development ending in seed dormancy |  |
| 2 | 264580_at | At1g05340 | hypothetical protein | AT1G05340 |  |  |  |
| 3 | 258499_at | At3g02540 | RAD23-3 (PUTATIVE DNA REPAIR PROTEIN RAD23-3); damaged DNA binding | RAD23-3 | proteasome binding///ubiquitin binding | nucleotide-excision repair/proteasomal ubiquitin-dependent protein catabolic process | nucleus |
| 4 | 258239_at | At3g27690 | LHCB2.3; chlorophyll binding | LHCB2.3 | chlorophyll binding | photosynthesis/response to blue light/response to far red light/response to red light | chloroplast envelope/chloroplast thylakoid membrane /thylakoid |
| 5 | 266462_at | At2g47770 | benzodiazepine receptor-related | AT2G47770 |  | response to abscisic acid stimulus/response to osmotic stress/response to salt stress | Golgi stack/endoplasmic reticulum/membrane |
| 6 | 258347_at | At3g17520 | late embryogenesis abundant domain-containing protein / LEA domain-containing protein | AT3G17520 |  | embryonic development ending in seed dormancy |  |
| 7 | 247095_at | At5g66400 | RAB18 (RESPONSIVE TO ABA 18) | RAB18 |  | cold acclimation/response to 1-aminocyclopropane-1-carboxylic acid/response to abscisic acid stimulus/response to stress/response to water deprivation |  |
| 8 | 247723_at | At5g59220 | protein phosphatase 2C, putative / PP2C, putative | AT5G59220 | catalytic activity/protein serine/threonine phosphatase activity | response to abscisic acid stimulus/response to water deprivation | chloroplast |
| 9 | 262382_at | At1g72920 | disease resistance protein (TIR-NBS class), putative | AT1G72920 | transmembrane receptor activity |  | intrinsic to membrane |
| 10 | 248352_at | At5g52300 | LTI65 (LOW-TEMPERATURE-INDUCED 65) | LTI65 |  | abscisic acid mediated signaling pathway/response to cold/response to salt stress/response to water deprivation |  |
| 11 | 247718_at | At5g59310 | LTP4 (LIPID TRANSFER PROTEIN 4); lipid binding | LTP4 | lipid binding | lipid transport/// response to abscisic acid stimulus/// response to water deprivation | endomembrane system |
| 12 | 257206_at | At3g16530 | legume lectin family protein | AT3G16530 | sugar binding |  | apoplast///cell wall///nucleus///plant-type cell wall |
| 13 | 264729_at | At1g22990 | heavy-metal-associated domain-containing protein / copper chaperone (CCH)-related | AT1G22990 | metal ion binding |  |  |
| 14 | 254189_at | At4g24000 | ATCSLG2; cellulose synthase/ transferase, transferring glycosyl groups | ATCSLG2 | cellulose synthase activity///transferase activity///transferase activity, transferring glycosyl groups | cellulose biosynthetic process///polysaccharide biosynthetic process | membrane |
| 15 | 245523_at | At4g15910 | ATDI21 (ARABIDOPSIS THALIANA DROUGHT-INDUCED 21) | ATDI21 |  | embryonic development///response to abscisic acid stimulus///response to water deprivation |  |
| 16 | 250648_at | At5g06760 | late embryogenesis abundant group 1 domain-containing protein / LEA group 1 domain-containing protein | AT5G06760 |  | embryonic development ending in seed dormancy |  |
| 17 | 247377_at | At5g63180 | pectate lyase family protein | AT5G63180 | lyase activity///pectate lyase activity |  |  |
| 18 | 253373_at | At4g33150 | lysine-ketoglutarate reductase/saccharopine dehydrogenase bifunctional enzyme | AT4G33150 | saccharopine dehydrogenase activity | L-lysine catabolic process | cytoplasm |
| 19 | 250942_at | At5g03350 | legume lectin family protein | AT5G03350 | sugar binding |  | apoplast///cell wall///chloroplast |
| 20 | 246487_at | At5g16030 | hypothetical protein | AT5G16030 |  |  |  |
| 21 | 251668_at | At3g57010 | strictosidine synthase family protein | AT3G57010 |  | biosynthetic process |  |
| 22 | 267559_at | At2g45570 | CYP76C2; electron carrier/ heme binding / iron ion binding / monooxygenase/ oxygen binding | CYP76C2 | electron carrier activity///heme binding///iron ion binding///monooxygenase activity///oxygen binding |  |  |
| 23 | 263544_at | At2g21590 | APL4; glucose-1-phosphate adenylyltransferase | APL4 | glucose-1-phosphate adenylyltransferase activity | starch biosynthetic process | glucose-1-phosphate adenylyltransferase complex |
| 24 | 265342_at | At2g18300 | basic helix-loop-helix (bHLH) family protein | AT2G18300 |  |  | nucleus |
| 25 | 260037_at | At1g68840 | RAV2(REGULATOR OF THE ATPASE OF THE VACUOLAR MEMBRANE); DNA binding/ transcription factor/ transcription repressor | RAV2 | DNA binding///transcription factor activity///transcription repressor activity | regulation of transcription, DNA-dependent | chloroplast |
| 26 | 249923_at | At5g19120 | aspartic-type endopeptidase | AT5G19120 | aspartic-type endopeptidase activity | proteolysis | endomembrane system |
| 27 | 264953_at | At1g77120 | ADH1 (ALCOHOL DEHYDROGENASE 1); alcohol dehydrogenase | ADH1 | alcohol dehydrogenase (NAD) activity///alcohol dehydrogenase (NAD) activity | cellular respiration///response to cadmium ion///response to hypoxia///response to osmotic stress///response to salt stress | cytosol///plasma membrane |
| 28 | 266578_at | At2g23910 | cinnamoyl-CoA reductase-related | AT2G23910 | binding///catalytic activity///coenzyme binding |  |  |
| 29 | 257271_at | At3g28007 | nodulin MtN3 family protein | AT3G28007 |  |  | endomembrane system///integral to membrane |
| 30 | 256245_at | At3g12580 | HSP70 (heat shock protein 70); ATP binding | HSP70 | ATP binding | protein folding///response to bacterium///response to cadmium ion///response to heat///response to high light intensity///response to hydrogen peroxide///response to virus | cell wall  ///cytosol///mitochondrion///plasma membrane |
| 31 | 258100_at | At3g23550 | MATE efflux family protein | AT3G23550 | antiporter activity///transporter activity |  | membrane |
| 32 | 264514_at | At1g09500 | cinnamyl-alcohol dehydrogenase family / CAD family | AT1G09500 |  | metabolic process |  |
| 33 | 262347_at | At1g64110 | AAA-type ATPase family protein | AT1G64110 | ATP binding///nucleotide binding |  |  |
| 34 | 251673_at | At3g57240 | BG3 (BETA-1,3-GLUCANASE 3); cellulase/ hydrolase, hydrolyzing O-glycosyl compounds | BG3 | cellulase activity///hydrolase activity, hydrolyzing O-glycosyl compounds | response to bacterium | apoplast///cell wall |
| 35 | 248236_at | At5g53870 | plastocyanin-like domain-containing protein | AT5G53870 | copper ion binding///copper ion binding///electron carrier activity |  | anchored to membrane///plasma membrane |
| 36 | 260287_at | At1g80440 | kelch repeat-containing F-box family protein | AT1G80440 |  |  |  |
| 37 | 250828_at | At5g05250 | hypothetical protein | AT5G05250 |  |  |  |
| 38 | 254234_at | At4g23680 | major latex protein-related / MLP-related | AT4G23680 |  |  |  |
| 39 | 264195_at | At1g22690 | gibberellin-responsive protein, putative | AT1G22690 |  | response to gibberellin stimulus |  |
| 40 | 251428_at | At3g60140 | DIN2 (DARK INDUCIBLE 2); catalytic/ cation binding / hydrolase, hydrolyzing O-glycosyl compounds | DIN2 | catalytic activity///cation binding///hydrolase activity, hydrolyzing O-glycosyl compounds///hydrolase activity, hydrolyzing O-glycosyl compounds | aging | endomembrane system |
| 41 | 249917_at | At5g22460 | esterase/lipase/thioesterase family protein | AT5G22460 | catalytic activity |  | plant-type cell wall |
| 42 | 251137_at | At5g01300 | phosphatidylethanolamine-binding family protein | AT5G01300 | phosphatidylethanolamine binding |  |  |
| 43 | 249626_at | At5g37540 | aspartyl protease family protein | AT5G37540 | aspartic-type endopeptidase activity | proteolysis | endomembrane system |
| 44 | 245463_at | At4g17030 | ATEXLB1 (ARABIDOPSIS THALIANA EXPANSIN-LIKE B1) | ATEXLB1 |  | plant-type cell wall loosening///sexual reproduction///unidimensional cell growth | endomembrane system///extracellular region |
| 45 | 265199_s_at | At2g36770 | UDP-glucoronosyl/UDP-glucosyl transferase family protein///UDP-glucoronosyl/UDP-glucosyl transferase family protein | AT2G36780///AT2G36770 | UDP-glycosyltransferase activity///transferase activity, transferring glycosyl groups///UDP-glycosyltransferase activity///transferase activity |  | /// |
| 46 | 262831_at | At1g14730 | hypothetical protein | AT1G14730 |  |  | endomembrane system///integral to membrane |
| 47 | 259786_at | At1g29660 | GDSL-motif lipase/hydrolase family protein | AT1G29660 | carboxylesterase activity |  |  |
| 48 | 259892_at | At1g72610 | GER1 (GERMIN-LIKE PROTEIN 1); oxalate oxidase | GER1 | NOT oxalate oxidase activity |  | extracellular matrix |
| 49 | 247684_at | At5g59670 | leucine-rich repeat protein kinase, putative | AT5G59670 |  | protein amino acid phosphorylation | endomembrane system |
| 50 | 249052_at | At5g44420 | PDF1.2 | PDF1.2 |  | defense response///defense response///jasmonic acid and ethylene-dependent systemic resistance///response to ethylene stimulus///response to insect///response to jasmonic acid stimulus///NOT response to salicylic acid stimulus | cell wall/// endomembrane system |
| 51 | 249039_at | At5g44310 | late embryogenesis abundant domain-containing protein / LEA domain-containing protein | AT5G44310 |  | embryonic development ending in seed dormancy |  |
| 52 | 251642_at | At3g57520 | AtSIP2 (Arabidopsis thaliana seed imbibition 2); hydrolase, hydrolyzing O-glycosyl compounds | AtSIP2 | hydrolase activity, hydrolyzing O-glycosyl compounds |  |  |
| 53 | 253293_at | At4g33905 | peroxisomal membrane protein 22 kDa, putative | AT4G33905 |  |  | peroxisomal membrane |
| 54 | 262482_at | At1g17020 | SRG1 (SENESCENCE-RELATED GENE 1); oxidoreductase, acting on diphenols and related substances as donors, oxygen as acceptor / oxidoreductase, acting on paired donors, with incorporation or reduction of molecular oxygen, 2-oxoglutarate as one donor, and inc | SRG1 | oxidoreductase activity, acting on diphenols and related substances as donors, oxygen as acceptor///oxidoreductase activity, acting on paired donors, with incorporation or reduction of molecular oxygen, 2-oxoglutarate as one donor, and incorporation of one atom each of oxygen into both donors | flavonoid biosynthetic process///organ senescence |  |
| 55 | 250826_at | At5g05220 | hypothetical protein | AT5G05220 |  |  |  |
| 56 | 250158_at | At5g15190 | hypothetical protein | AT5G15190 |  |  |  |
| 57 | 260264_at | At1g68500 | hypothetical protein | AT1G68500 |  |  |  |
| 58 | 260974_at | At1g53440 | leucine-rich repeat family protein / protein kinase family protein | AT1G53440 |  | protein amino acid phosphorylation | plasma membrane |
| 59 | 256114_at | At1g16850 | hypothetical protein | AT1G16850 |  | response to salt stress |  |
| 60 | 254823_at | At4g12580 | hypothetical protein | AT4G12580 |  |  |  |
| 61 | 262047_at | At1g80160 | lactoylglutathione lyase family protein / glyoxalase I family protein | AT1G80160 | lactoylglutathione lyase activity | carbohydrate metabolic process |  |
| 62 | 249860_at | At5g22860 | serine carboxypeptidase S28 family protein | AT5G22860 |  | proteolysis | endomembrane system |
| 63 | 260357_at | At1g69260 | AFP1 (ABI FIVE BINDING PROTEIN) | AFP1 |  | abscisic acid mediated signaling pathway | nucleus |
| 64 | 254660_at | At4g18250 | receptor serine/threonine kinase, putative | AT4G18250 | transmembrane receptor protein serine/threonine kinase activity |  |  |
| 65 | 259161_at | At3g01500 | CA1 (CARBONIC ANHYDRASE 1); carbonate dehydratase/ zinc ion binding | CA1 | carbonate dehydratase activity///carbonate dehydratase activity///zinc ion binding | defense response to bacterium///defense response to fungus, incompatible interaction///regulation of stomatal movement///response to carbon dioxide///response to cold | apoplast///chloroplast thylakoid membrane///membrane///stromule///thylakoid |
| 66 | 262307_at | At1g71000 | heat shock protein binding | AT1G71000 |  | protein folding |  |
| 67 | 254250_at | At4g23290 | protein kinase family protein | AT4G23290 | kinase activity | protein amino acid phosphorylation | mitochondrion |
| 68 | 261077_at | At1g07430 | protein phosphatase 2C, putative / PP2C, putative | AT1G07430 | catalytic activity///protein serine/threonine phosphatase activity///protein serine/threonine phosphatase activity | protein amino acid dephosphorylation | protein serine/threonine phosphatase complex |
| 69 | 258321_at | At3g22840 | ELIP1 (EARLY LIGHT-INDUCABLE PROTEIN); chlorophyll binding | ELIP1 | chlorophyll binding | response to cold |  |
| 70 | 248505_at | At5g50360 | hypothetical protein | AT5G50360 |  |  |  |
| 71 | 259705_at | At1g77450 | anac032 (Arabidopsis NAC domain containing protein 32); transcription factor | anac032 | transcription factor activity | multicellular organismal development///regulation of transcription |  |
| 72 | 248164_at | At5g54490 | PBP1 (PINOID-BINDING PROTEIN 1); calcium ion binding / protein binding | PBP1 | calcium ion binding///protein binding | response to auxin stimulus |  |
| 73 | 249894_at | At5g22580 | hypothetical protein | AT5G22580 |  |  |  |
| 74 | 255344_s_at | At4g04540 | protein kinase family protein///protein kinase family protein | AT4G04570///AT4G04540 | kinase activity///kinase activity | protein amino acid phosphorylation///protein amino acid phosphorylation | endomembrane system///endomembrane system |
| 75 | 256796_at | At3g22210 | hypothetical protein | AT3G22210 |  |  |  |
| 76 | 253494_at | At4g31830 | hypothetical protein | AT4G31830 |  |  |  |
| 77 | 253344_at | At4g33550 | lipid binding | AT4G33550 | lipid binding | lipid transport |  |
| 78 | 247061_at | At5g66780 | hypothetical protein | AT5G66780 |  |  |  |
| 79 | 254809_at | At4g12410 | auxin-responsive family protein | AT4G12410 |  | response to auxin stimulus |  |
| 80 | 251928_at | At3g53980 | protease inhibitor/seed storage/lipid transfer protein (LTP) family protein | AT3G53980 | lipid binding | lipid transport |  |
| 81 | 261957_at | At1g64660 | ATMGL (ARABIDOPSIS THALIANA METHIONINE GAMMA-LYASE); catalytic/ methionine gamma-lyase | ATMGL | catalytic activity///methionine gamma-lyase activity | cellular amino acid metabolic process///methionine catabolic process via 2-oxobutanoate | cytosol |
| 82 | 259364_at | At1g13260 | RAV1; DNA binding / transcription factor/ transcription repressor | RAV1 | DNA binding/// transcription factor activity///transcription repressor activity | lateral root development///leaf development///negative regulation of flower development///regulation of transcription, DNA-dependent///response to brassinosteroid stimulus | nucleus/nucleus |
| 83 | 260877_at | At1g21500 | hypothetical protein | AT1G21500 |  |  | 1-phosphatidylinositol-4-phosphate 3-kinase, class IA complex |
| 84 | 267080_at | At2g41190 | amino acid transporter family protein | AT2G41190 | amino acid transmembrane transporter activity | amino acid transport | membrane |
| 85 | 258139_at | At3g24520 | AT-HSFC1; DNA binding / transcription factor | AT-HSFC1 | DNA binding///transcription factor activity | regulation of transcription, DNA-dependent | nucleus |
| 86 | 266098_at | At2g37870 | protease inhibitor/seed storage/lipid transfer protein (LTP) family protein | AT2G37870 | lipid binding | lipid transport | endomembrane system |
| 87 | 252487_at | At3g46660 | UGT76E12 (UDP-GLUCOSYL TRANSFERASE 76E12); UDP-glycosyltransferase/ quercetin 3-O-glucosyltransferase/ quercetin 7-O-glucosyltransferase | UGT76E12 | UDP-glycosyltransferase activity///quercetin 3-O-glucosyltransferase activity///quercetin 7-O-glucosyltransferase activity///transferase activity, transferring glycosyl groups | metabolic process |  |
| 88 | 260380_at | At1g73870 | zinc finger (B-box type) family protein | AT1G73870 |  |  | intracellular |
| 89 | 255891_at | At1g17870 | EGY3 (ETHYLENE-DEPENDENT GRAVITROPISM-DEFICIENT AND YELLOW-GREEN-LIKE 3) | EGY3 |  | response to heat///response to high light intensity///response to hydrogen peroxide | chloroplast |
| 90 | 249174_at | At5g42900 | hypothetical protein | AT5G42900 |  | response to cold |  |
| 91 | 247487_at | At5g62150 | peptidoglycan-binding LysM domain-containing protein | AT5G62150 |  | cell wall macromolecule catabolic process |  |
| 92 | 253657_at | At4g30110 | HMA2; cadmium-transporting ATPase | HMA2 | cadmium-transporting ATPase activity | ATP biosynthetic process///metabolic process///metal ion transport | endomembrane system///integral to membrane///membrane |
| 93 | 266503_at | At2g47780 | rubber elongation factor (REF) protein-related | AT2G47780 |  |  |  |
| 94 | 258764_at | At3g10720 | pectinesterase, putative | AT3G10720 | enzyme inhibitor activity///pectinesterase activity | cell wall modification | endomembrane system |
| 95 | 252123_at | At3g51240 | F3H (FLAVANONE 3-HYDROXYLASE); naringenin 3-dioxygenase | F3H | naringenin 3-dioxygenase activity | flavonoid biosynthetic process///flavonoid biosynthetic process///response to UV-B |  |
| 96 | 252515_at | At3g46230 | ATHSP17.4 | ATHSP17.4 |  | response to heat |  |
| 97 | 258880_at | At3g06420 | ATG8H (autophagy 8h); microtubule binding | ATG8H | microtubule binding | autophagy |  |
| 98 | 249454_at | At5g39520 | hypothetical protein | AT5G39520 |  |  |  |
| 99 | 265024_at | At1g24600 | hypothetical protein | AT1G24600 |  |  |  |
| 100 | 260143_at | At1g71880 | SUC1 (Sucrose-proton symporter 1); carbohydrate transmembrane transporter/ sucrose:hydrogen symporter/ sugar:hydrogen symporter | SUC1 | carbohydrate transmembrane transporter activity///sucrose:hydrogen symporter activity///sucrose:hydrogen symporter activity///sugar:hydrogen symporter activity | pollen germination///response to nematode | membrane///plasma membrane///plasma membrane///vacuole |
